# Small Nucleolar RNAs as Emerging Players in Cancer Biology and Precision Medicine

**DOI:** 10.3390/cancers17233847

**Published:** 2025-11-30

**Authors:** Hans Kristian Lorenzo

**Affiliations:** 1 INSERM UMR-S-MD 1197, 94803 Villejuif, France; hans.lorenzo@inserm.fr; 2 Faculté de Médecine, Université Paris-Saclay, 94270 Le Kremlin-Bicêtre, France; 3 Department of Nephrology, Bicêtre Hospital, AP-HP, 94270 Le Kremlin-Bicêtre, France

**Keywords:** small nucleolar RNA, cancer biology, ribosomal dysfunction, biomarkers, non-coding RNA, therapeutic targets

## Abstract

Small nucleolar RNAs (snoRNAs) constitute a class of non-coding RNAs traditionally associated with ribosomal RNA modification. However, recent research has revealed that these molecules participate in a wide range of cellular processes with significant implications for cancer biology. This review examines the emerging evidence on snoRNA dysregulation in various tumor types, where they can function as both oncogenes and tumor suppressors. We analyze the multiple molecular mechanisms through which snoRNAs contribute to malignant transformation, including the generation of specialized ribosomes, alternative splicing regulation, epigenetic modulation, and post-transcriptional regulatory functions. We discuss the potential of snoRNAs as non-invasive biomarkers for cancer diagnosis and prognosis, as well as emerging strategies for their utilization as therapeutic targets. The integration of these findings positions snoRNAs as fundamental players in oncogenesis and opens new avenues for the development of clinical tools in precision cancer medicine.

## Simple Summary

Small nucleolar RNAs were long considered simple molecular guides for ribosome assembly. However, these tiny molecules participate in far more cellular processes than previously imagined, with profound implications for cancer development. This review synthesizes current evidence showing that these molecules can act as both cancer promoters and suppressors, influencing how cells translate genetic information into proteins, regulate gene expression, and respond to therapy. We examine how their abnormal levels appear across virtually all cancer types and discuss their emerging potential as blood-based diagnostic tests that could detect cancer early without invasive procedures. Understanding these molecules opens new possibilities for developing both diagnostic tools to identify cancer patients who will benefit most from specific treatments and novel therapies that target these molecules directly, representing a promising avenue for personalized cancer medicine.

## 1. Introduction

For over five decades, small nucleolar RNAs have occupied a seemingly specialized niche in cell biology. These molecules of 60 to 300 nucleotides, initially discovered in the 1960s as components of small nucleolar ribonucleoprotein complexes, were long considered secondary players in the context of gene regulation. Their primary function appeared clear and limited: guiding chemical modifications of ribosomal RNA within the nucleolus, where ribosomes are assembled. However, as frequently occurs in molecular biology, this simplistic view has given way to a much more nuanced and fascinating understanding of these small yet powerful molecules.

**Figure 1:**
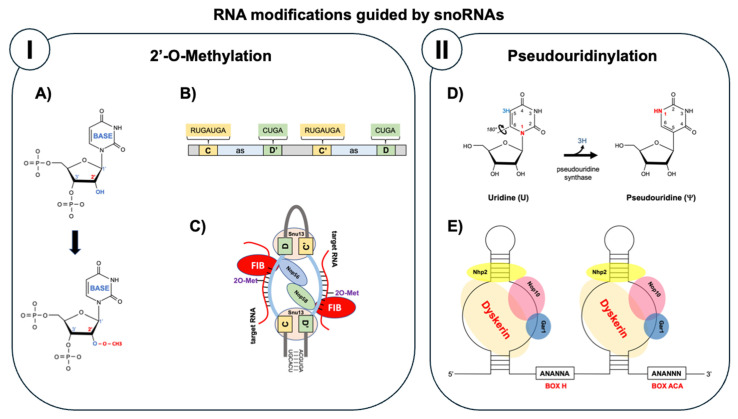
Canonical functions of snoRNAs. Panel (I): C/D box snoRNAs guide 2′-O-methylation of target RNAs. (**A**) Conversion of 2′-OH to 2′-O-CH_3_. (**B**) Box C/D snoRNA structure with conserved motifs and antisense guide regions. (**C**) Box C/D snoRNP complex with fibrillarin (methyltransferase, FIB), Nop56, Nop58, and Snu13. Panel (II): H/ACA box snoRNAs guide pseudouridylation of target RNAs. (**D**) Isomerization of uridine to pseudouridine through 180° base rotation. (**E**) Box H/ACA snoRNA structure. H/ACA snoRNP complex with dyskerin (pseudouridine synthase), Gar1, Nop10, and Nhp2.

As shown in Figure 1, snoRNAs are classified into two main families based on their conserved sequence motifs and the type of chemical modification they guide (Kiss, 2002; Matera et al., 2007) [1,2]. Box C/D snoRNAs (also designated as SNORDs) contain characteristic C (RUGAUGA) and D (CUGA) box motifs near their 5′ and 3′ ends, respectively, with internal C’ and D’ boxes often present. These snoRNAs guide 2′-O-methylation, a modification where a methyl group (CH_3_) is added to the 2′ hydroxyl position of the ribose sugar, converting the 2′-OH to 2′-O-CH_3_. This methylation occurs precisely 5 nucleotides upstream from the D or D’ box, with substrate specificity determined by 10–21 nucleotide antisense elements that form Watson–Crick base pairs with the target RNA. The box C/D small nucleolar RNA–protein complex (snoRNP complex) assembles with four essential proteins: fibrillarin (the methyltransferase enzyme), Nop56, Nop58, and Snu13 (15.5K in humans), which together position the catalytic site for precise modification (Figure 1) [3].

Box H/ACA snoRNAs (designated as SNORAs) possess a distinct bipartite structure consisting of two hairpins connected by a single-stranded hinge region containing the conserved H box (ANANNA), terminating with an ACA box precisely 3 nucleotides before the 3′ end. These snoRNAs guide pseudouridylation, an isomerization reaction that converts uridine to pseudouridine (Ψ) by breaking the N-glycosidic bond and rotating the uracil base 180° to form a C-glycosidic bond. This modification creates an additional hydrogen bond donor, enhancing RNA stability and affecting RNA structure. The target uridine is identified through complementary sequences in internal loops called pseudouridylation pockets, positioned exactly 14–16 nucleotides from either the H or ACA box. The H/ACA snoRNP machinery comprises four core proteins: dyskerin (the pseudouridine synthase), Gar1, Nop10, and Nhp2, which form a stable complex that catalyzes this isomerization (Figure 1) [1,2].

While most snoRNAs localize to the nucleolus where ribosome biogenesis occurs, a subset called small Cajal body-specific RNAs (scaRNAs) concentrate in Cajal bodies, nuclear structures involved in snRNP maturation. scaRNAs guide modifications of spliceosomal snRNAs and can contain both C/D and H/ACA domains within a single molecule, enabling them to catalyze both types of modifications. The trafficking of snoRNAs between the nucleolus and nucleoplasm is mediated by specific localization signals and protein interactions, with some snoRNAs showing dynamic localization patterns in response to cellular conditions.

The human genome harbors approximately 400 snoRNA genes, a number that continues to expand as next-generation sequencing techniques reveal previously unnoticed transcripts. The snoDB database has systematically cataloged these genes and their tissue expression patterns, revealing unexpected complexity [4]. Many snoRNAs show highly specific expression profiles that do not always correlate with those of their host genes, suggesting sophisticated post-transcriptional regulatory mechanisms. This tissue specificity acquires particular relevance in the cancer context, where altered snoRNA expression patterns emerge as distinctive molecular signatures of different tumor types.

The revolution in our understanding of snoRNAs began with the discovery of their non-canonical functions. Pioneering studies by Kishore and colleagues (2010) demonstrated that certain snoRNAs can be processed into smaller fragments capable of regulating alternative splicing [5]. Subsequently, it was discovered that some snoRNAs participate in mRNA stabilization, modulate chromatin structure, and can even function analogously to microRNAs, regulating gene expression at the post-transcriptional level [6,7,8]. This functional versatility has transformed our perception of snoRNAs from simple modification guides to multifaceted regulators capable of influencing numerous aspects of cellular physiology.

The link between snoRNAs and cancer has strengthened considerably in recent years. The accumulated evidence indicates that dysregulation of specific snoRNAs is not merely a passive consequence of malignant transformation, but an active participant in oncogenesis. Different snoRNAs can function as oncogenes or tumor suppressors, and their altered expression levels correlate with important clinical features such as tumor stage, metastatic capacity, and treatment response [9,10,11]. This association has opened new perspectives both for understanding the fundamental mechanisms of cancer and for developing innovative diagnostic and therapeutic tools.

This review integrates mechanistic, diagnostic, and therapeutic perspectives on snoRNA involvement in cancer, providing a synthesis that bridges these traditionally separate research areas. We propose that understanding snoRNA molecular functions should be directly connected with their potential as biomarkers and therapeutic targets, rather than considering these applications in isolation. By examining the relationships between snoRNA biology and cancer pathogenesis alongside their diagnostic and therapeutic possibilities, we aim to provide a more cohesive view of how these small regulatory molecules contribute to cancer and how this knowledge can be translated into clinical applications. This integrated approach highlights both opportunities and challenges that emerge when considering snoRNAs across the full spectrum from basic biology to potential medical applications.

## 2. Dysregulation of snoRNAs in Cancer

The alteration in snoRNA expression patterns constitutes a recurring feature in virtually all cancer types studied to date. However, this dysregulation is far from being a random or chaotic phenomenon. On the contrary, consistent patterns emerge that suggest specific functional roles for different snoRNAs in tumor biology. The complexity of these patterns reflects the inherent heterogeneity of cancer and underscores the need to understand snoRNAs not as isolated entities, but as components integrated into broader regulatory networks.

### 2.1. Oncogenic snoRNAs

Among the snoRNAs that actively promote malignant transformation and progression, several have been characterized in sufficient detail to understand their mechanisms of action. Some examples are examined here (see Table 1). For example, SNORA21 illustrates how snoRNAs can function as molecular oncogenes. In colorectal cancer, SNORA21 exhibits significantly elevated expression levels and actively participates in the modulation of multiple cancer-related signaling pathways, including the Hippo, Wnt, and Axon guidance pathways, which collectively regulate cell cycle progression, proliferation, and invasion [12]. Importantly, this oncogenic activity appears to extend beyond canonical rRNA modification mechanisms, suggesting that SNORA21 influences these signaling cascades through non-traditional functional mechanisms, thereby illustrating the expanding repertoire of snoRNA functions in cancer biology.

Another case is SNORD78, which provides interesting insights into how alteration of rRNA modifications can contribute to oncogenesis. This snoRNA, consistently overexpressed in colorectal tumors, directs hypermethylation of specific sites in 28S rRNA [13]. These aberrant modifications create ribosomes with altered translational properties that favor the synthesis of proteins encoded by mRNAs with particular structural features in their 5′ untranslated regions. Many oncogenes and growth factors possess precisely these characteristics, resulting in their preferential translation by these modified “oncoribosomes” [14,15]. In recent work, Lan and colleagues have elucidated additional aspects of this mechanism, demonstrating that cancer cells can actively remodel their translational machinery to favor pro-tumoral gene expression programs [13].

The case of SNORD16 emerged as a particularly relevant prognostic biomarker in digestive tract cancers [16]. Several studies established that SNORD16 overexpression not only correlates with adverse clinical features such as increased tumor size and presence of lymph node metastases but functions as an independent prognostic factor [16]. At the molecular level, SNORD16 promotes cell proliferation and inhibits apoptosis through mechanisms involving both its canonical function in rRNA modification and non-canonical effects on specific mRNA stability [16]. The situation is further complicated by the fact that SNORD16 resides within the lncRNA SNHG16, which also possesses independent oncogenic functions. This nested genomic architecture creates complex regulatory circuits where the host lncRNA and embedded snoRNA can act synergistically to promote the malignant phenotype [17].

A recent discovery of particular clinical relevance is the role of SNORA47 in Luminal A breast cancer. SNORA47 is not only overexpressed in this breast cancer subtype but actively contributes to two critical cancer characteristics: the maintenance of tumor stemness and chemotherapy resistance. The underlying mechanism involves a previously uncharacterized regulatory axis including transcription factor EBF3, ribosomal protein RPL11, and the oncogene c-Myc [18]. This molecular cascade directly connects snoRNA expression with regulation of one of the most important oncogenes in cancer biology, providing a clear example of how snoRNAs can influence critical nodes of tumor signaling networks.

### 2.2. Tumor Suppressor snoRNAs

At the other extreme of the functional spectrum, several snoRNAs have been demonstrated to act as molecular guardians against malignant transformation (Table 1). SNORA24 exemplifies this protective role through its essential function in maintaining translational fidelity. This snoRNA guides pseudouridylation at U609 and U863 within 18S rRNA, modifications that are critical for accurate ribosome decoding. When SNORA24 expression is reduced or lost, ribosomes lacking these modifications exhibit compromised translational accuracy, resulting in increased coding errors that accumulate progressively and contribute to proteomic instability. In a hepatocellular carcinoma model driven by oncogenic RAS, McMahon and colleagues demonstrated that this loss of translational fidelity enables cells to bypass oncogene-induced senescence, thereby allowing tumor development and validating SNORA24 as tumor suppressor [19].

SNORD44 represents another molecular guardian whose loss facilitates tumor progression. This C/D box snoRNA, encoded within the GAS5 lncRNA, exhibits downregulated expression in colorectal cancer and is subject to p53-dependent regulation. Yuan and colleagues demonstrated that therapeutic restoration of SNORD44 expression via an oncolytic adenovirus effectively suppressed colorectal tumor growth both in vitro and in vivo, with enhanced efficacy when combined with rapamycin treatment. These findings established SNORD44 as a tumor suppressor whose loss compromises cellular growth arrest mechanisms [20].

Another important feature to note is the potential tissue specificity of snoRNAs. An example of this can be found in SNORD113-1. This snoRNA shows markedly reduced expression specifically in hepatocellular carcinoma, where it normally functions by inhibiting the MAPK/ERK and STAT3 signaling pathways [21]. In this regard, Xu and colleagues (2014) demonstrated that the loss of SNORD113-1 directly contributes to hepatocarcinogenesis by allowing the uncontrolled activation of these proliferative pathways. Furthermore, experimental restoration of SNORD113-1 in hepatocarcinoma cell lines suppresses their growth and reduces their tumorigenic capacity, confirming its role as a liver-specific tumor suppressor [21].

Other additional snoRNAs have also emerged as critical tumor suppressors across different cancer types. SNORD50A and SNORD50B, located at chromosome 6q14.3, represent particularly compelling examples, as they are recurrently deleted in 10–40% of twelve common cancer types, with their loss associated with reduced patient survival. These snoRNAs directly bind to K-Ras protein and inhibit its oncogenic activity by preventing farnesyltransferase-mediated activation and reducing GTP-bound active K-Ras levels, thereby suppressing hyperactivation of the Ras-ERK signaling pathway [22]. Functional studies have demonstrated that SNORD50A/B undergo frequent genomic deletions and transcriptional downregulation in both prostate and breast cancers, with specific mutations showing significant associations with clinically aggressive disease [23,24]. Similarly, SNORD47 demonstrates tumor suppressive properties in glioma by suppressing cell proliferation, inducing G2 phase arrest, and inhibiting invasion and epithelial-mesenchymal transition, with low expression correlating with advanced tumor stage and poor overall survival [25]. Together, these snoRNAs exemplify the diverse mechanisms through which small nucleolar RNAs can act as molecular guardians against malignant transformation. Importantly, their downregulation in cancer suggests that restoring their expression or mimicking their molecular functions may represent a viable therapeutic strategy.

### 2.3. Context-Dependent Functional Plasticity

Perhaps one of the most intriguing aspects of snoRNA biology in cancer is the observation that some of these RNAs can function as oncogenes or tumor suppressors depending on cellular context. SNORD76 exemplifies this remarkable functional duality. In hepatocellular carcinoma, SNORD76 functions as an oncogene, with its expression significantly upregulated in tumor tissues and correlating with poorer patient survival [26,27]. It promotes tumorigenesis through activation of the Wnt/β-catenin pathway, enhanced cell proliferation, and induction of epithelial-mesenchymal transition [27]. In contrast, the same SNORD76 molecule exhibits tumor suppressor properties in glioblastoma, where it is selectively downregulated in WHO grade IV tumors and its enforced expression inhibits proliferation by inducing cell cycle arrest at S phase [25,28]. Several mechanisms may underlie this plasticity, including tissue-specific availability of binding partners, differential activation of signaling pathways across cancer types, and distinct epigenetic landscapes. This functional duality has critical therapeutic implications: strategies targeting snoRNAs must be carefully tailored to specific cancer types and molecular contexts, as depleting a snoRNA acting as an oncogene in one cancer could paradoxically promote tumor growth in another malignancy where it functions as a tumor suppressor [29,30]. Additionally, SNORA13 acts as a tumor suppressor by inducing cellular senescence through direct interaction with ribosomal protein RPL23, which impairs 60S ribosomal subunit assembly and triggers p53 activation [31]. This mechanism links ribosome biogenesis surveillance to tumor suppression through senescence induction.

**Table 1:** Representative snoRNAs with validated oncogenic or tumor suppressor functions in human cancers. This table summarizes key snoRNAs discussed in the text, their molecular mechanisms, and validation methods. HCC: hepatocellular carcinoma.

snoRNA	Type	Function	Cancer Type	Primary Mechanism	Validation	Reference
SNORA21	H/ACA	Oncogene	Colorectal	Hippo/Wnt pathways	In vitro/in vivo	[13]
SNORD78	C/D	Oncogene	Colorectal	Oncoribosome formation	Patient samples	[13]
SNORA47	H/ACA	Oncogene	Breast	EBF3/RPL11/c-Myc axis	Xenografts	[18]
SNORA24	H/ACA	Tumor suppressor	HCC	Translational fidelity	RAS model	[11]
SNORD44	C/D	Tumor suppressor	Colorectal	p53 pathway	Oncolytic virus	[20]
SNORD113-1	C/D	Tumor suppressor	HCC	MAPK/STAT3 inhibition	In vitro/in vivo	[21]
SNORA13	H/ACA	Tumor suppressor	Multiple	Senescence via RPL23/p53	Cell models	[31]
SNORD50A/B	C/D	Context-dependent	Multiple	K-Ras/TRIM21	Xenografts	[22,23,24]
SNORD76	C/D	Context-dependent	HCC/Glioblastoma	Wnt/Cell cycle	Patient tissues	[25,27,28]

## 3. Molecular Mechanisms of snoRNA-Mediated Effects in Cancer

**Figure 2:**
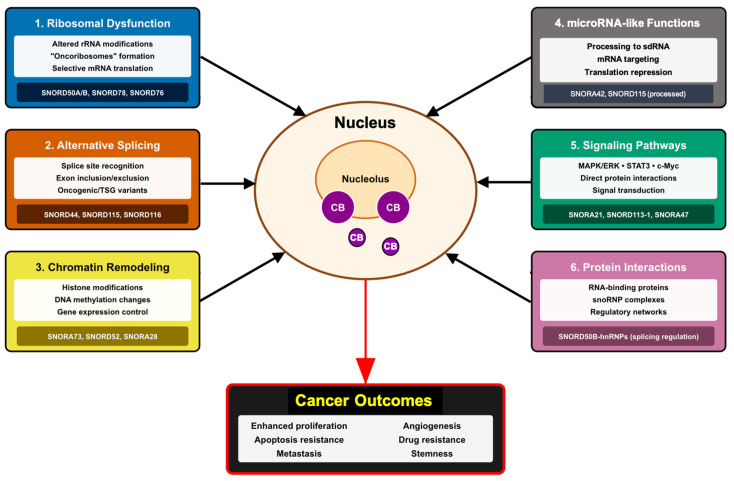
Integration of snoRNA-mediated mechanisms in cancer biology. The diagram illustrates the convergence of multiple pathways through which dysregulated snoRNAs contribute to the malignant phenotype. Mechanisms include: (1) generation of oncoribosomes with selective translational capacity, (2) alteration of alternative splicing patterns, (3) epigenetic remodeling of the genome, (4) production of sdRNAs with regulatory functions, (5) direct modulation of oncogenic signaling pathways, and (6) formation of regulatory complexes with RNA-binding proteins. These mechanisms act synergistically to promote cancer hallmarks including uncontrolled proliferation, apoptosis evasion, invasive capacity, angiogenesis, and therapeutic resistance. CB: Cajal Bodies.

SnoRNAs drive cancer progression through multifaceted molecular mechanisms spanning ribosomal dysfunction, post-transcriptional gene regulation, epigenetic control, microRNA-like functions, and integration with cellular signaling networks through dynamic protein interactions (Figure 2). The main mechanisms are summarized in Table 2, which provides an integrated overview of representative snoRNAs, their cancer-related functions, and associated molecular pathways.

### 3.1. Ribosomal Dysfunction and Translational Control

The concept of specialized ribosomes or “onco-ribosomes” represents an emerging paradigm in contemporary cancer biology. For decades, ribosomes were considered uniform translation machines. However, experimental evidence now demonstrates that cells generate ribosomes with distinct modification patterns that exhibit selective translational preferences [40]. In vivo CRISPR screens and ribosome profiling have revealed that the differential expression of ribosomal proteins like RPL15 creates specialized ribosomes that selectively translate specific mRNA subsets, promoting metastatic progression [41].

SnoRNAs play a central role in this specialization by directing site-specific 2′-O-methylations and pseudouridylations that alter ribosomal structure and function, influencing which mRNAs are preferentially translated [14]. Cancer cells actively exploit this mechanism to remodel their proteome without need for additional genetic changes. For instance, SNORD78 overexpression in colorectal cancer does not simply result in “more” rRNA methylation, but in specific methylation patterns that create preferential binding sites for certain mRNAs. These mRNAs, which incidentally encode many proteins related to proliferation, survival, and metastasis, are more efficiently translated by these modified ribosomes [42,43]. Nait Slimane and colleagues (2020) has extensively documented this phenomenon, demonstrating that oncoribosomes can selectively increase translation of oncogenes while maintaining or even reducing synthesis of tumor suppressor proteins [15]. Ribosomal heterogeneity extends beyond simple changes in translational efficiency. snoRNA-directed modifications can affect translation fidelity, reading frame selection, and even the ribosome’s capacity for translational reinitiation. These effects have profound consequences for cellular homeostasis and can contribute to multiple cancer hallmarks, from apoptosis evasion to metabolic reprogramming [15].

### 3.2. Alternative Splicing Regulation

Pre-mRNA alternative splicing is a fundamental process that allows cells to generate multiple proteins from a single gene. In cancer, splicing patterns are frequently altered, producing protein isoforms that promote tumor growth, invasion, and therapy resistance [44]. snoRNAs have emerged as important regulators of this process, acting through mechanisms that extend beyond their canonical functions.

The capacity of snoRNAs to modulate alternative splicing was first demonstrated by Kishore and Stamm (2006) in the context of Prader-Willi syndrome, where they showed that the orphan snoRNA HBII-52 regulates alternative splicing of the serotonin receptor 2C [45]. Subsequently, Bazeley and colleagues (2008) used computational approaches to demonstrate that many orphan snoRNAs localize near alternative splicing sites across the genome, suggesting a broader regulatory role for this class of molecules [46]. This observation was later confirmed by detailed mechanistic studies showing that snoRNAs can be processed into smaller fragments that directly interact with the spliceosome and modulate splicing site selection [5].

The loss of SNORD44 in various cancer types illustrates the pathological consequences of snoRNA-mediated splicing dysregulation. Under normal conditions, SNORD44 promotes inclusion of exons encoding important functional domains in tumor suppressor proteins, while suppressing exon inclusion in oncogenes that would result in more active isoforms [20]. Its absence in cancer cells reverses these patterns, creating a protein repertoire that favors tumor proliferation and survival. This mechanism affects not only individual genes but can remodel entire splicing networks, thus amplifying its impact on the cellular phenotype.

In addition to influencing canonical alternative splicing, snoRNAs may also be linked to backsplicing and circRNA biogenesis through specific structural and genomic contexts. Several studies have demonstrated that introns containing snoRNA genes often possess repetitive or complementary sequences that promote RNA base pairing and bring splice sites into close proximity, facilitating exon circularization (Liang & Wilusz, 2014; PMID: 25281217) [47]. Moreover, transcripts hosting large snoRNA clusters, such as SNORD115 and SNORD116, produce abundant non-linear RNA species, including circular forms, and these snoRNA-rich regions have been shown to modulate RNA processing and stability in a manner compatible with circRNA production (Falaleeva et al., 2015; PMID: 26220404) [48]. Although not directly cancer-related, the Prader-Willi syndrome locus exemplifies how snoRNA clusters can generate abundant circular transcripts in neuronal tissues, providing proof-of-principle for mechanisms that may also operate in cancer contexts where both snoRNAs and circRNAs are frequently dysregulated [49]. Together, these data suggest that snoRNAs and snoRNA-containing introns may contribute to the architectural conditions that enable circRNA formation, providing an additional layer of RNA processing regulation relevant to cancer.

### 3.3. Chromatin Remodeling and Epigenetic Regulation

SnoRNA participation in epigenetic regulation represents a significant expansion beyond their canonical nucleolar functions, establishing new links between RNA metabolism and genome organization. Several snoRNAs demonstrate the capacity to interact with chromatin and modulate its accessibility through distinct mechanisms, thereby influencing gene expression at transcriptional and post-transcriptional levels. The orphan snoRNA SNORA73 exemplifies non-canonical chromatin interaction in acute myeloid leukemia (AML). This snoRNA localizes to chromatin where it forms a non-canonical ribonucleoprotein complex with PARP1 and the canonical H/ACA proteins DKC1/NHP2 at DNA damage sites. SNORA73 inhibits PARP1 auto-PARylation, thereby blocking DNA damage repair and maintaining the genome instability characteristic of hematopoietic malignancies [37].

SnoRNA-derived RNAs (sdRNA) further expand this epigenetic repertoire. The sdnRNA3, expressed in melanoma tumor-associated macrophages (TAMs), represses Nos2 gene transcription by recruiting the chromatin-remodeling regulator Mi-2β and depositing the repressive histone mark H3K27me3 at the gene promoter, thereby reducing chromatin accessibility and promoting an immunosuppressive tumor microenvironment [38]. Complementing this direct chromatin-remodeling mechanism, SNORD104 modulates epigenetic regulation in endometrial cancer through 2′-O-methylation of PARP1 mRNA, increasing transcript stability and protein levels of this polymerase involved in DNA repair and chromatin structure modification [36].

These mechanisms illustrate that snoRNAs and their derivatives transcend their canonical nucleolar roles to function as epigenetic regulators through diverse strategies: from direct recruitment of chromatin-remodeling complexes and histone modification to indirect modulation of key enzymes governing chromosomal architecture.

### 3.4. MicroRNA-like Functions

The discovery that some snoRNAs can function analogously to microRNAs has revealed a completely new dimension in the biology of these RNAs. This phenomenon involves the regulated processing of snoRNAs into smaller fragments, termed sdRNAs (snoRNA-derived RNAs), which incorporate into the RISC complex and regulate gene expression post-transcriptionally [50]. Like canonical microRNAs, sdRNAs operate through 6–8 nucleotide “seed” regions that mediate specific binding to the 3′UTR regions of target mRNAs [51]. However, sdRNAs exhibit distinctive properties in terms of molecular stability, subcellular localization, and diversity of target genes. Recent studies suggest that this processing is not accidental, but can be induced by specific conditions such as cellular stress or oncogenic signals [52].

Several sdRNAs have demonstrated significant functional roles in different cancer types. SNORA42 generates sdRNAs that target multiple oncogene mRNAs, functioning as endogenous tumor suppressors [34]. Conversely, sdRNA-93 promotes invasion in breast cancer, particularly in Luminal B HER2+ tumors, by directly regulating PIPOX [33]. Similarly, SNORD78 and its derived sdRNAs are significantly elevated in patients with metastatic disease in lung and prostate cancer [35].

Some snoRNAs exhibit dual functionality, simultaneously fulfilling their canonical roles in rRNA modification and generating functional sdRNAs in a context-dependent manner. Pan-cancer analyses of TCGA data have identified hundreds of snoRNAs associated with clinical stage and patient survival [10], and sdRNAs show more pronounced expression changes than microRNAs in certain tumor contexts [35]. Notably, many microRNA discovery algorithms have erroneously discarded small RNAs that align with snoRNAs, suggesting that the actual repertoire of post-transcriptional regulators relevant to cancer is substantially larger than currently appreciated. Recent investigations by Huo et al. (2024) have initiated the systematic cataloging of sdRNAs and their roles in cancer, revealing that these RNAs represent an emerging class of regulators with potential as biomarkers and therapeutic targets [8]. Given their similarity to microRNAs in biogenesis and function, sdRNAs add an additional layer of complexity to snoRNA-mediated oncogenic programs.

### 3.5. Integration with Signaling Networks

snoRNAs do not operate in isolation but integrate deeply into cellular signaling networks. This integration occurs at multiple levels, from modulation of key signaling pathway components to regulation of pathway gene expression. For example, SNORA21 has been identified as a key oncogenic snoRNA in colorectal cancer, where its inhibition results in decreased cell proliferation and invasion through modulation of multiple cancer-related pathways, including the Hippo and Wnt signaling cascades [12]. Similarly, other snoRNAs directly impact critical oncogenic pathways: SNORD60 regulates the PI3K/AKT/mTOR pathway by enhancing the stability and expression of PIK3CA mRNA through 2′-O-methylation, thereby promoting cancer progression [32]. This multilayered integration allows snoRNAs to influence not only the activity of signaling pathways but also the expression levels and stability of their key components, positioning them as important modulators of cellular signaling networks in cancer. Likewise, the recent discovery of the SNORA47/EBF3/RPL11/c-Myc axis in breast cancer illustrates how snoRNAs can connect different levels of cellular regulation [18]. SNORA47 influences expression of transcription factor EBF3, which in turn regulates ribosomal protein RPL11. RPL11, beyond its structural function in the ribosome, can sequester MDM2 and stabilize p53, but also affects c-Myc stability and function. This cascade connects ribosomal biogenesis with two of the most important regulators of cell growth and apoptosis, providing multiple potential therapeutic intervention points [18].

### 3.6. snoRNA–Protein Interactions and Regulatory Complexes

In addition to forming canonical snoRNP particles with core proteins such as fibrillarin, NOP56 and NOP58, snoRNAs engage in a wide range of protein interactions that substantially expand their functional repertoire in cancer [53]. These interactions allow snoRNAs to operate not only as RNA-guided modification enzymes but also as molecular adaptors, scaffolds, and regulatory cofactors that integrate distinct layers of RNA and protein homeostasis. As a result, snoRNA–protein complexes can influence diverse processes including ribosome specialization, stress responses, splicing regulation, chromatin remodeling, and signaling pathway activation [42].

Canonical snoRNA–protein assemblies play well-established roles in rRNA and snRNA modification. Aberrant expression of specific box C/D or box H/ACA snoRNAs can alter the stoichiometry or activity of these complexes, leading to changes in 2′-O-methylation or pseudouridylation that remodel ribosome architecture. Such changes can selectively enhance the translation of oncogenic mRNAs, contributing to malignant phenotypes. However, it is increasingly clear that many oncogenic functions arise from non-canonical snoRNA–protein interactions, which enable snoRNAs to act outside their classical nucleolar environment. Among the best-characterized examples are SNORD50A and SNORD50B, which directly bind K-Ras. Loss of these snoRNAs increases the pool of active GTP-loaded Ras and enhances downstream ERK1/2 signaling, with recurrent deletions found in 10–40% of pancreatic, colorectal, and lung cancers [22]. The same snoRNAs have a second, mechanistically distinct role in breast cancer: they facilitate the formation of a TRIM21–GMPS complex, promoting p53 degradation and tumor progression specifically in p53 wild-type backgrounds [39]. This functional duality—tumor suppressive through inhibition of Ras signaling in some tissues, and oncogenic through TRIM21–GMPS stabilization in others—illustrates the deep context dependence of snoRNA–protein interactions in cancer [54]. Such tissue-specific effects reflect variations in protein availability, chromatin state, and stress conditions that shape the composition and activity of snoRNA-associated regulatory complexes.

Stress-induced snoRNA–protein interactions provide another important dimension of snoRNA biology in cancer. Under metabolic stress, the rpL13a-encoded snoRNAs U32a, U33 and U35a relocalize from the nucleolus to the cytoplasm, where they associate with stress-response proteins and contribute to pro-survival pathways [52]. Similarly, transcriptional inhibition remodels nucleolar organization and drives snoRNP components into nucleolar caps, reflecting a dynamic redistribution of snoRNA-bound complexes that may be co-opted by cancer cells to withstand fluctuations in nutrient or oxygen availability [55]. These observations highlight the plasticity of snoRNA–protein interactions and suggest that tumor cells may exploit these stress-adapted assemblies to support growth under adverse microenvironmental conditions.

More recently, high-resolution interactomic and chimeric eCLIP analyses have uncovered previously unrecognized snoRNA-binding partners, revealing that snoRNA-containing ribonucleoproteins are more heterogeneous and functionally specialized than previously appreciated. Accessory factors such as WDR43 and NOLC1 modulate snoRNA target selection and influence both ribosome and spliceosome biogenesis, forming regulatory hubs with roles extending beyond RNA modification [56]. A striking example is SNORD89, which directs 2′-O-methylation at two adjacent positions in U2 snRNA, fine-tuning splice-site recognition. Dysregulation of SNORD89 alters splicing programs and promotes endometrial tumorigenesis by modulating 2′-O-methylation of Bim, thereby affecting apoptotic pathways [57]. These findings underscore how individual snoRNAs can act as highly specific modulators of protein-RNA complexes with consequences for cell fate, stress response, and oncogenic signaling.

Collectively, these data reveal that snoRNAs function as integral components of multifaceted protein networks whose composition and activity are dynamically shaped by cellular state and tumor context. The diversification of snoRNA-associated complexes—ranging from canonical snoRNPs to stress-responsive assemblies and newly defined nucleoplasmic regulatory units—illustrates the breadth of mechanisms through which snoRNAs influence cancer biology.

## 4. snoRNAs as Cancer Biomarkers

Translation of fundamental knowledge about snoRNAs toward clinical applications represents one of the most promising developments in this field. snoRNAs possess several characteristics that make them ideal biomarkers: they are stable in biological fluids, show tissue- and tumor-type specific expression patterns, and their levels can be measured with high sensitivity and specificity using established technologies.

### 4.1. Diagnostic Applications

The diagnostic potential of snoRNAs has been demonstrated across multiple cancer types through diverse biological specimens, each offering distinct advantages for clinical implementation. Circulating biomarkers represent one of the most accessible approaches for widespread screening (see Table 3). Li and colleagues (2024) identified a four-snoRNA panel (SNORD16, SNORA73B, SCARNA4, and SNORD49B) for breast cancer detection in plasma, achieving 74.38% specificity and 66.51% sensitivity. These snoRNAs exhibited remarkable stability, resisting RNase A treatment, and were predominantly found in vesicle-free plasma fractions, simplifying extraction procedures compared to exosome-dependent biomarkers [58]. Complementing this plasma-based approach, exosomal snoRNAs have proven particularly valuable for addressing specific diagnostic challenges. In lung cancer, Cao and colleagues (2024) validated that serum exosomal SNORD78 and SNORD37 can accurately distinguish malignant from benign pulmonary nodules, a critical clinical need that could reduce unnecessary biopsies while improving early detection of malignancy [59]. The differential stability and compartmentalization of snoRNAs in various blood fractions thus provides multiple strategies for biomarker development depending on the clinical context.

Beyond blood-based approaches, snoRNA detection in other biological fluids has significantly expanded non-invasive screening capabilities. In colorectal cancer, Gómez-Matas and colleagues (2024) demonstrated that fecal SNORA51, combined with hemoglobin concentration, significantly improved diagnostic accuracy among individuals with positive fecal immunochemical tests, enhancing the specificity of population screening strategies [60]. This refinement of screening protocols addresses a major challenge in colorectal cancer detection: reducing false-positive rates that lead to unnecessary colonoscopies. Similarly, urinary biomarkers offer another accessible non-invasive avenue. Zhou and colleagues (2023) identified a three-snoRNA signature (SNORD15A, SNORD35B, and SNORD60) in renal cell carcinoma, demonstrating significant upregulation in tumor tissues alongside stable detection in urinary sediment, thus providing a practical monitoring tool for kidney malignancies [61]. Tissue-based snoRNA profiling has complemented these fluid biomarker studies, with SCARNA12 emerging as a promising diagnostic marker in colorectal cancer tissue samples [62] and comprehensive profiling revealing distinctive signatures in head and neck squamous cell carcinomas [63]. Together, these findings across diverse cancer types and specimen sources demonstrate the remarkable versatility of snoRNAs as diagnostic and prognostic biomarkers, establishing them as promising tools for cancer detection and patient stratification in clinical oncology.

**Table 3:** Non-invasive snoRNA biomarker panels for cancer diagnosis. Performance metrics are shown where available. AUC: area under the curve; FIT: fecal immunochemical test; N/A: not available.

Cancer Type	snoRNA Panel	Sample Type	Sensitivity	Specificity	AUC	Reference
Breast	SNORD16, SNORA73B, SCARNA4, SNORD49B	Plasma	66.5%	74.4%	N/A	[58]
Lung	SNORD78, SNORD37	Serum exosomes	N/A	N/A	0.85	[59]
Colorectal	SNORA51	Fecal	82%	89%	0.91	[60]
Renal cell	SNORD15A, SNORD35B, SNORD60	Urine sediment	78%	85%	0.88	[61]
HCC	9-snoRNA signature	Tissue	N/A	N/A	0.92	[64]

### 4.2. Prognostic Value and Disease Monitoring

Beyond their diagnostic applications, snoRNAs provide valuable prognostic information that can guide therapeutic decisions and predict clinical outcomes in cancer patients. Specific snoRNA expression patterns have been consistently associated with adverse clinical features including advanced tumor stage, metastatic spread, and reduced overall survival across diverse malignancy types. The prognostic utility of these molecules has been particularly well-demonstrated through the development of multi-snoRNA signatures that can stratify patients into distinct risk groups with superior predictive performance compared to conventional clinicopathological staging systems. In hepatocellular carcinoma, for instance, a 9-snoRNA signature developed through multivariate Cox regression analysis serves as an independent prognostic factor with enhanced predictive accuracy [64]. Similarly, in breast cancer, the integration of snoRNA profiles with other molecular biomarkers has yielded promising results, with SNORA47 correlating with advanced tumor stage and unfavorable survival outcomes in luminal A subtype patients [18], while a comprehensive 13-snoRNA signature has demonstrated independent prognostic value in multivariate analysis, effectively stratifying patients for both overall survival and recurrence-free survival [65]. This stratification capability proves particularly critical for therapeutic decision-making in borderline cases where the benefit of adjuvant chemotherapy remains uncertain.

The clinical relevance of snoRNAs as prognostic biomarkers extends prominently to hematological malignancies, where distinct expression profiles have been characterized across multiple disease subtypes. Comprehensive analyses of acute leukemias have revealed distinctive snoRNA signatures in both myeloid and lymphoblastic variants, with acute promyelocytic leukemia exhibiting a particularly specific expression pattern [66]. In chronic lymphocytic leukemia, the analysis of 211 patients established a two-snoRNA prognostic model incorporating SNORA70F and SNORD116-18 that successfully distinguished distinct prognostic groups independent of traditional markers such as IGHV mutational status [67]. The prognostic significance of snoRNAs in lymphoid malignancies extends further to peripheral T-cell lymphoma, where HBII-239 expression correlates significantly with prolonged progression-free and overall survival in patient cohorts [68].

In solid tumors, snoRNAs have emerged as equally valuable prognostic markers with clinical applicability across multiple cancer types. Non-small cell lung cancer studies have identified several snoRNAs—including SNORA47, SNORA68, and SNORA78—as independent predictors of poor prognosis after adjustment for clinical parameters [69], while the expression of SNORA3 and SNORA42 in tumor-initiating cells shows inverse correlation with patient survival [70]. In the colorectal cancer setting, SNORA42 has been established as an independent prognostic factor for both overall survival and disease-free survival, with particular utility in identifying high-risk patients for recurrence among those with stage II disease [71]. The widespread clinical relevance of snoRNAs across malignancies has been further validated through pan-cancer analyses examining more than 10,000 samples from 31 cancer types, which identified 355 snoRNAs significantly associated with patient survival [10].

The integration of computational approaches has enhanced the discovery and validation of prognostic snoRNA signatures. Machine learning algorithms applied to snoRNA expression patterns across eight major cancer types have successfully identified discriminative molecules including HBII-52-14, HBII-336, and SNORD123 with demonstrated prognostic value, illustrating the potential of computational methods to systematically extract clinically relevant information from complex expression datasets [72].

## 5. snoRNAs as Therapeutic Targets

Growing evidence linking snoRNAs to essential oncogenic processes has raised the possibility that these molecules may represent therapeutically actionable nodes rather than biomarkers alone. Although targeting RNA species located in the nucleus—and particularly within the nucleolus—poses specific challenges, recent technological advances have considerably expanded the feasibility of modulating snoRNA abundance or function in cancer.

Antisense oligonucleotides (ASOs) constitute the most direct and mature therapeutic modality for targeting snoRNAs. Their ability to enter the nucleus, accumulate efficiently, and recruit RNase H makes them suitable for degrading snoRNA precursors or blocking snoRNP assembly. Chemical backbone modifications such as phosphorothioate linkages, 2′-O-methoxyethyl (2′-MOE) groups, and gapmer architectures overcome many of the barriers imposed by the stable secondary structures of snoRNAs. Proof-of-principle studies have shown that ASO-mediated inhibition of SNORD50A/B can attenuate tumor growth in p53 wild-type models by blocking the TRIM21–GMPS axis and restoring p53 function [39]. Hu and colleagues (2024) have comprehensively reviewed ASO-based therapeutic strategies targeting snoRNAs in cancer, discussing both preclinical successes and challenges remaining for clinical translation [53]. Sophisticated designs must consider not only the target snoRNA sequence but also its structure and molecular context. Computational tools such as PLEXY, developed by Kehr and colleagues (2011), have significantly facilitated prediction of box C/D snoRNA targets, allowing more rational design of therapeutic interventions [73]

Genome-editing approaches further expand the therapeutic landscape. Most snoRNAs in mammals are located within introns of protein-coding or non-coding host genes [74], making selective deletion technically challenging. Nevertheless, recent work has demonstrated that individual snoRNAs can be precisely disrupted without altering neighboring snoRNAs or compromising host gene function [74]. Such studies provide strong proof that snoRNA-specific editing is achievable. Beyond DNA editing, CRISPR–Cas13 systems enable direct RNA targeting and degradation, offering a reversible and highly specific approach to modulate oncogenic snoRNAs or their sdRNA derivatives without affecting the underlying genome [75].

Indirect targeting strategies provide an additional layer of therapeutic opportunities. Epigenetic drugs such as DNA methyltransferase inhibitors can restore the expression of tumor-suppressive snoRNAs silenced by promoter hypermethylation, as seen for SNORD123, U70C, and ACA59B in colorectal and lung cancers [76]. Small-molecule inhibitors of ribosome biogenesis—such as CX-5461 or BMH-21—may also interfere with nucleolar pathways in which oncogenic snoRNAs are embedded, reducing their functional output and dampening specialized ribosome formation [55]. Furthermore, disrupting interactions between snoRNAs and key snoRNP proteins (e.g., fibrillarin, NOP56, NOP58) represents a promising but still underexplored approach, given the essential role of these interactions in snoRNA stability and function [75].

Importantly, therapeutic modulation of snoRNAs does not require direct physical access to the nucleolus, addressing a central concern commonly raised when considering the druggability of nucleolar processes. A substantial proportion of snoRNA-mediated oncogenic functions occur outside the nucleolus—including regulation of alternative splicing, mRNA stability, chromatin remodeling and cytoplasmic stress responses [5,18,20,32,33].

Importantly, therapeutic modulation of snoRNAs does not necessary require direct physical access to the nucleolus. A substantial proportion of snoRNA-mediated oncogenic activities occur outside the nucleolus, including the regulation of alternative splicing by orphan snoRNAs and sdRNA-derived fragments [50], the stabilization of oncogenic transcripts through targeted 2′-O-methylation [36], and the modulation of chromatin states via PARP1-associated complexes [19]. In addition, specific snoRNAs relocalize to the cytoplasm under stress conditions, where they exert pro-survival functions [52]. Together, these findings demonstrate that many oncogenic effects of snoRNAs arise in the nucleoplasm or cytoplasm, reinforcing the idea that their therapeutic targeting does not depend on direct nucleolar penetration. Targeting these pathways can therefore attenuate snoRNA-driven oncogenic programs without necessitating nucleolar penetration. Moreover, chemically modified antisense oligonucleotides accumulate efficiently in the nucleus and can degrade nuclear RNAs, including snoRNA precursors [50,52] Nuclear RNA-targeting strategies, such as RNA editing or RNA-level disruption of intronic snoRNA sequences, have also been shown to be feasible without affecting host gene architecture [75]. Together, these mechanistic and technological advances counter the notion that snoRNAs should be viewed solely as biomarkers and instead support their potential as functionally relevant and therapeutically actionable molecules.

Therapeutic relevance is further underscored by the observation that several snoRNAs influence treatment response. For instance, SNORD33 predicts platinum sensitivity in triple-negative breast cancer [77], while SNORA47 drives chemoresistance through the EBF3/RPL11/c-Myc axis [18]. SnoRNA-mediated regulation of immune evasion pathways also points to potential synergies between snoRNA-directed therapies and immunotherapies. The combined ability of snoRNAs to shape oncogenic signaling, regulate drug sensitivity, and modulate immune responses suggests that they may serve as valuable components of multimodal therapeutic strategies.

Despite the promise of snoRNA-directed therapies, several limitations remain. These include challenges in achieving tissue-specific delivery, distinguishing highly similar snoRNA family members, quantifying snoRNAs accurately across platforms, and avoiding off-target effects when manipulating intronic host-gene architecture. Additionally, the incomplete annotation of human snoRNA families requires more comprehensive transcriptomic and functional studies, as highlighted by recent large-scale efforts to refine ncRNA catalogs [78]. Addressing these gaps will require standardized pipelines, reference materials and systematic validation in multicenter cohorts. Nevertheless, the convergence of RNA therapeutics, genome and transcriptome editing, and nucleolar biology strongly suggests that snoRNA-targeted interventions are both technically feasible and biologically justified, firmly establishing snoRNAs as more than passive biomarkers and positioning them as emerging therapeutic targets in cancer.

## 6. Conclusions and Perspectives

The field of snoRNAs in cancer stands at a critical juncture between fundamental discovery and clinical translation. Over the past two decades, these molecules have evolved from simple rRNA modification guides into recognized regulators of cancer biology, participating in virtually all aspects of oncogenesis—from translational machinery reprogramming through specialized ribosomes to modulation of signaling networks and epigenetic landscapes. This accumulated evidence demonstrates their profound involvement in tumorigenesis, yet transforming these insights into clinical applications presents both formidable challenges and unprecedented opportunities that define the current state of the field.

The path toward clinical implementation confronts intertwined technical and biological obstacles that together shape the translational landscape. Accurate detection of snoRNAs remains problematic due to their stable secondary structures interfering with conventional extraction and amplification methods, while their biological complexity—simultaneously participating in multiple cellular processes—makes predicting consequences of their modulation difficult. The context-dependent duality observed with specific snoRNAs exemplifies this challenge particularly well. SNORA24 functions as a tumor suppressor in RAS-driven hepatocellular carcinoma by maintaining ribosomal translation fidelity [19], while SNORD50A and SNORD50B demonstrate opposing roles depending on p53 status, acting as oncogenes in p53 wild-type breast cancers through TRIM21-GMPS-mediated p53 degradation but functioning as tumor suppressors in other contexts [39]. This functional plasticity underscores the necessity for cancer-type-specific and molecular context-specific therapeutic strategies. Tumor heterogeneity compounds these difficulties, as different cellular populations may show distinct dependencies on specific snoRNAs, potentially leading to heterogeneous therapeutic responses.

However, these very complexities are driving innovation in ways that promise to overcome current limitations. Emerging single-cell sequencing technologies are beginning to reveal dynamic snoRNA expression patterns during tumor progression and therapy response at unprecedented resolution, while artificial intelligence and machine learning approaches are accelerating discovery of clinically relevant snoRNA signatures that capture biological complexity impossible to detect through traditional analyses [72]. Development of international consortia and comprehensive databases like snoDB, recently enhanced as snoDB 2.0 with improved snoRNA-RNA target prediction and expression profiling capabilities, will prove crucial for standardizing methodologies, sharing data across diverse cancer types, and accelerating clinical translation [4,79].

Progress toward clinical implementation has been particularly notable for biomarkers, where circulating snoRNA panels demonstrate diagnostic capacity comparable or superior to conventional markers. The therapeutic potential of snoRNAs, though in earlier developmental stages, is accelerating through advances in oligonucleotide technology, delivery systems, and genomic editing. As the field matures, snoRNAs are positioned to occupy an increasingly prominent place in precision cancer medicine, bridging fundamental cell biology with clinical applications and opening new avenues for clinical translation.

As we enter an era where RNA-based diagnostics and targeted therapeutics are becoming clinical reality, snoRNAs stand poised to transform cancer medicine, offering the possibility that within the next decade, comprehensive snoRNA profiling could guide personalized treatment decisions from initial diagnosis through therapeutic monitoring, fundamentally reshaping how we detect, classify, and treat cancer.

## Figures and Tables

**Table 2:** Major oncogenic mechanisms through which dysregulated snoRNAs contribute to cancer development and progression. ASO: antisense oligonucleotides; IRES: internal ribosome entry site.

Oncogenic Mechanism	snoRNA Examples	Cancer Effect	Therapeutic Potential	References
**Ribosomal Dysfunction**				
Aberrant 2′-O-methylation	SNORD78, SNORD60	Selective oncogene translation	ASO targeting	[13,32]
Loss of pseudouridylation	SNORA24	Reduced translational fidelity	Expression restoration	[19]
Oncoribosomes	SNORD16	IRES-mediated translation	Ribosome inhibitors	[17]
**Post-transcriptional Regulation**				
MicroRNA-like functions	sdRNA-93, SNORA42	Target mRNA regulation	sdRNA inhibitors	[16,33,34]
Alternative splicing	SNORD44, SNORD115 (HBII-52)	Pro-tumoral isoforms	Splicing modulators	[5,20,35]
mRNA stability	SNORD104	Enhanced PARP1 expression	PARP inhibitors	[36]
**Chromatin Remodeling**				
PARP1 interaction	SNORA73	Genomic instability	PARP inhibitors	[37]
Histone modification	sdnRNA3	TAM immunosuppression	Epigenetic therapy	[38]
**Signaling Networks**				
Oncogenic pathways	SNORA21, SNORD113-1	Proliferation/survival	Combination therapy	[12,21]
Protein interactions	SNORD50A/B	K-Ras activation	Targeted inhibitors	[22,39]

## Abbreviations

*AAV*	Adeno-associated virus
*AML*	Acute myeloid leukemia
*ASO*	Antisense oligonucleotide
*AUC*	Area under the curve
*cfRNA*	Cell-free RNA
*CLL*	Chronic lymphocytic leukemia
*CRC*	Colorectal cancer
*CTC*	Circulating tumor cell
*DKC1*	Dyskerin
*EMT*	Epithelial-mesenchymal transition
*EVs*	Extracellular vesicles
*FBL*	Fibrillarin
*FIT*	Fecal immunochemical test
*HCC*	Hepatocellular carcinoma
*IGHV*	Immunoglobulin heavy chain variable region
*lncRNA*	Long non-coding RNA
*LNA*	Locked nucleic acid
*MM*	Multiple myeloma
*ncRNA*	Non-coding RNA
*NSCLC*	Non-small cell lung cancer
*RNP*	Ribonucleoprotein
*ROC*	Receiver operating characteristic
*ROS*	Reactive oxygen species
*sdRNA*	snoRNA-derived RNA
*siRNA*	Small interfering RNA
*snoRNA*	Small nucleolar RNA
*snoRNP*	Small nucleolar ribonucleoprotein
*TCGA*	The Cancer Genome Atlas
